# The sensitivity of qSOFA calculated at triage and during emergency department treatment to rapidly identify sepsis patients

**DOI:** 10.1038/s41598-020-77438-8

**Published:** 2020-11-23

**Authors:** Sarah M. Perman, Mark E. Mikkelsen, Munish Goyal, Adit Ginde, Abhishek Bhardwaj, Byron Drumheller, S. Cham Sante, Anish K. Agarwal, David F. Gaieski

**Affiliations:** 1 Department of Emergency Medicine, University of Colorado School of Medicine, Aurora, USA; 2 Division of Pulmonary and Critical Care Medicine, Perelman School of Medicine at the University of Pennsylvania, Philadelphia, USA; 3 Departments of Emergency and Critical Care Medicine, MedSTAR Washington Hospital Centre, Washington, USA; 4 Division of Critical Care Medicine, Cleveland Clinic Department of Internal Medicine, Cleveland, USA; 5 Department of Emergency Medicine, Albert Einstein Medical Center, Philadelphia, USA; 6 Department of Emergency Medicine, University of New Mexico School of Medicine, Philadelphia, USA; 7 Department of Emergency Medicine, Perelman School of Medicine at the University of Pennsylvania, Philadelphia, USA; 8 Department of Emergency Medicine, Vice Chair for Resuscitation Services, Director of Emergency Critical Care, Enterprise Physician Lead for Sepsis Care, Sidney Kimmel Medical College at Thomas Jefferson University, 1025 Walnut Street; 300 College Building, Philadelphia, PA 19107 USA

**Keywords:** Biomarkers, Medical research, Risk factors, Signs and symptoms

## Abstract

The quick sequential organ failure assessment (qSOFA) score has been proposed as a means to rapidly identify adult patients with suspected infection, in pre-hospital, Emergency Department (ED), or general hospital ward locations, who are in a high-risk category with increased likelihood of “poor outcomes:” a greater than 10% chance of dying or an increased likelihood of spending 3 or more days in the ICU. This score is intended to replace the use of systemic inflammatory response syndrome (SIRS) criteria as a screening tool; however, its role in ED screening and identification has yet to be fully elucidated. In this retrospective observational study, we explored the performance of triage qSOFA (tqSOFA), maximum qSOFA, and first initial serum lactate (> 3 mmol/L) at predicting in-hospital mortality and compared these results to those for the initial SIRS criteria obtained in triage. A total of 2859 sepsis cases were included and the in-hospital mortality rate was 14.4%. The sensitivity of tqSOFA ≥ 2 and maximum qSOFA ≥ 2 to predict in-hospital mortality were 33% and 69%, respectively. For comparison, the triage SIRS criteria and the initial lactate > 3 mmol/L had sensitivities of 82% and 65%, respectively. These results demonstrate that in a large ED sepsis database the earliest measurement of end organ impairment, tqSOFA, performed poorly at identifying patients at increased risk of mortality and maximum qSOFA did not significantly outperform initial serum lactate levels.

## Introduction

Sepsis, the syndrome of the body’s pathophysiologic response to infection, is common and deadly^1–3^. Early identification and timely initiation of treatment for patients with sepsis^4–7^ have resulted in an improvement in morbidity and overall mortality from sepsis^8–11^. During the decades in which these improvements in sepsis understanding, recognition, and care have occurred, sepsis Quality Improvement Initiatives relied initially on the 1991 ACCP/SCCM Consensus Conference definition of severe sepsis, which was subsequently modified in 2001 at the International Sepsis Definitions Conference^12,13^. This definition of severe sepsis included the presence of two or more systemic inflammatory response syndrome (SIRS) criteria, a documented or suspected infection, and at least one acute, infection-related organ dysfunction. In 2016 the Third International Consensus Definitions for Sepsis and Septic Shock (SEPSIS-3) were published, which redefined sepsis as “life-threatening organ dysfunction caused by a dysregulated host response to infection.” SEPSIS-3 also eliminated the use of the SIRS criteria, which were considered neither sufficiently sensitive nor specific, in the definition of sepsis^14^. SEPSIS-3 further eliminated the concept of severe sepsis, opting instead to redefine sepsis as infection plus acute organ dysfunction and septic shock as sepsis requiring vasopressors in the setting of an elevated lactate^1^.

The SEPSIS-3 authors suggested that “In out-of-hospital, emergency department, or general hospital ward settings, adult patients with suspected infection can be rapidly identified as being more likely to have poor outcomes” by applying a rapid bedside assessment score, known as the “quick Sequential [Sepsis-related] Organ Failure Assessment (qSOFA) score^1^,” consisting of a respiratory rate of 22 or more breaths per minute, altered mentation, and a systolic blood pressure of 100 mmHg or less. The SEPSIS-3 authors suggested this should be calculated to facilitate early recognition of sepsis in patients at high risk of poor outcomes (increased mortality or increased length of ICU stay). Large administrative data analyzed by the SEPSIS-3 authors suggested that a qSOFA score of ≥ 2 would rapidly identify non-ICU patients “more likely to have poor outcomes typical of sepsis,” defined as in-hospital mortality > 10%, with an area under the receiver operating characteristic (AUROC) curve of 0.81 (compared to 0.76 for the SIRS criteria; *p* = 0.01)^1,15^. The authors concluded that the new definitions should “facilitate earlier recognition and more timely management of patients with sepsis or at risk of developing sepsis^1^.” Since this assertion in 2016, numerous authors have analyzed the usefulness of qSOFA in retrospective and prospective cohorts at different points in the care continuum from pre-hospital^16,17^ to initial triage^18–20^ to the period of ED management^20,21^ to in-patient wards and the ICU^15^; have looked at it as a screening tool for all patients presenting to the ED^22^ or for those with suspected infection^23,24^; have investigated dynamic changes in qSOFA during ED stay^20,25^; have analyzed its accuracy as a predictor of ICU admission, length of stay, and in-hospital mortality^26^; have tried to improve the performance of qSOFA by add various biomarkers including lactate^27,28^, procalcitonin^29^, monocyte distribution width^30^, and CRP combined with mid-regional proadrenomedullin^31^ or vital sign measures including heart rate variability^32^, EtCO_2_^33^, and shock index^19^; have examined its utility in high and low resource settings^27,29,34^; and have compared it to other scoring systems including SIRS, MEWS, NEWS, and conventional SOFA^35,36^. All of these studies provide important clinical information and have various limitations mainly related to the data sets used, the presence or absence of serial qSOFA values, the clinical setting where the studies were performed, and the overall mortality of the cohorts. Because of this, a comprehensive, generally agreed upon strategy for early screening, identification, and initiation of time-sensitive treatment for sepsis patients remains elusive.

In this retrospective cohort study, we utilized a large data set of patients with *known sepsis and a high rate of in-hospital mortality* and sought to determine the sensitivity of qSOFA to rapidly identify patients who present to the ED, have two or more SIRS criteria during their ED stay, a documented or suspected source of infection, an acute sepsis-related organ dysfunction, and are admitted to the hospital, either to the wards or the ICU. In other words, when these patients are first seen in the ED and are undifferentiated patients does the use of qSOFA have early applicability to capture a significant majority of patients previously captured by SIRS criteria? First, we calculated the triage qSOFA (tqSOFA)–derived from triage vital signs at the time of ED presentation–in order to determine the ability of qSOFA to capture critically ill patients as early as possible upon presentation for care. Second, we calculated the maximum qSOFA obtained during the entire ED stay to analyze whether an iterative, composite measure of qSOFA accounting for the worst variables during a sepsis patient’s initial management course would be more predictive of outcome. We calculated the sensitivity, specificity, and area under the receive operator characteristic (AUROC) curve of tqSOFA, maximum qSOFA, and initial venous lactate > 3 mmol/L, which was the cut-off for organ dysfunction in the 2^nd^ International Sepsis Definitions^14^, at identifying sepsis patients at risk for increased in-hospital mortality.

## Methods

### Study design

This was a retrospective, observational cohort study of consecutive patients admitted from the ED with severe sepsis present on admission, with treatment initiated in the ED, according to the 1992 and 2001 consensus conference definitions^12,13^. The data were collected from a large, urban, teaching hospital, with an ED annual volume of 60,000 to 65,000 visits per year, 750 in-patient beds, and 150 intensive care unit beds, between September 9, 2003 and December 31, 2009. Data were manually abstracted from the patient record by trained research assistants and stored as de-identified data on a secure server at the University of Pennsylvania. This study was approved by the Thomas Jefferson University Office of Human Research’s Institutional Review Board, was granted a waiver of informed consent, and all methods were carried out in accordance with relevant guidelines and regulations.

### Study population

Consecutive adult patients presenting to the ED and admitted to the hospital were screened for inclusion in the database. Adults ≥ 18 years old with severe sepsis were identified in a stepwise fashion. Suspected infection was identified by triage chief complaints consistent with infection, orders for blood cultures, collection of culture specimens, and administration of antibiotics. Patients were categorized as “severe sepsis” if the medical record exhibited the presence of ≥ 2 SIRS criteria (counted cumulatively over the ED stay), a documented or suspected source of infection, and the presence of at least one acute organ dysfunction, including a lactate > 3 mmol/L, as defined by the 2001 consensus definitions^13^. For consistency with the 2016 SEPSIS-3 definitions, in this manuscript the patients identified as having “severe sepsis” for the purposes of inclusion in the database will be referred to as “sepsis patients” (patients with a source of infection and acute, life-threatening organ dysfunction).

### Data collection

Beginning January 1, 2005, trained research assistants surveyed all admitted patients from the daily logs of the hospital’s electronic medical record (EMR) as part of a comprehensive sepsis quality improvement project that included lactate screening^4^, early antibiotic administration^37^, and initiation of protocolized care in the most critically ill subset of patients^8^. For benchmarking purposes, a cohort of historic controls was also collected from patients presenting between September 9, 2003 and December 31, 2004. If the patient was admitted to the hospital but screened as SIRS negative, orders were reviewed for blood cultures, other cultures, and antibiotic orders in an attempt to avoid exclusion of SIRS negative severe sepsis patients.

Clinical data collected from the ED EMR included patient demographics, initial, serial and worst vital signs, initial Glasgow coma score, comorbidities, interventions with time stamps indicating when they were initiated (intravenous fluids, antibiotics, vasopressors, EGDT, intubation), laboratory values, imaging studies, length of stay, and admission location. From the hospital’s in-patient EMR, further data were obtained including culture results and sensitivities, hospital length of stay, length of time on ventilator, in-hospital mortality, and discharge location.

For this research question, we utilized data points available in the database to calculate tqSOFA defined as the qSOFA score present when the patient was triaged in the ED, and maximum qSOFA during the ED stay. Beginning January 1, 2005, an initial whole blood venous lactate was introduced into ED practice as a screening tool for potential sepsis patients, to further risk stratify the severity of sepsis, and to help guide resuscitation efficacy.

### Statistical analysis

For descriptive analysis, continuous data are expressed as means (± standard deviations) and differences were tested using Student’s t-test. Categorical variables are presented as percentages and analyzed using the Chi-squared test or Fischer’s exact method. Sensitivity, specificity, negative predicted value and positive predicted value were determined for each of the three tests (tqSOFA, qSOFA, lactate > 3 mmol/L) at the previously determined cut-point for the test to be predictive of mortality (tqSOFA and qSOFA ≥ 2, and lactate > 3 mmol/L). A c-statistic was determined as a measure of the AUROC curve (AUROC: 0.6–0.7, poor; 0.7–0.8, adequate; 0.8–0.9, good; and 0.9 to 1, excellent)^37^, for each cut-point (STATA, version 14.1, College Station, TX).

### Ethical approval

Institutional Review Board approval was obtained and this work was granted waiver of informed consent.

## Results

### Demographics

Mean age of the 2859 patients was 57.3 ± 17.8 years, 52.9% were male, and 47.3% were African-American (Table 1). Seventy-two percent (2059/2859) of the cohort were admitted to the ICU at some point during their hospital stay, 8.5% (241/2859) were intubated in the ED; 7.2% (205/2859) of patients required vasopressor support during their ED stay; the mean time to administration of appropriate antibiotics was 184 ± 157 min and the mean fluid volume administrated during the ED stay was 2460 ± 1759 mL. In-hospital mortality was 14.4%, 28 day mortality was 19.0%, and mean ED length of stay (LOS) was 477 min (± 248 min). The in-hospital mortality for patients who spent time in the ICU was 16.7%; for those who were never admitted to the ICU it was 8.4%.

**Table 1:** Patient Demographics and clinical characteristics stratified by serial qSOFA values.

Variables (n)	All patients (2859)	Triage qSOFA < 2 (2337)	Triage qSOFA ≥ 2 (508)	*p*-value	Maximum qSOFA < 2 (1478)	Maximum qSOFA ≥ 2 (1362)	*p*-value
Age (yr)	57.3 ± 17.8	56.2 ± 17.7	62.2 ± 17.8	< 0.01	54.8 ± 17.7	60.0 ± 17.6	< 0.01
Male	52.9% (1522)	53% (1243)	51% (261)	ns	54.3% (802)	51.4% (700)	ns
Time to antibiotic (min)	184 ± 157	197 ± 162	125 ± 114	< 0.01	204 ± 167	162 ± 141	< 0.01
Total IVF (mL)	2460 ± 1759	2405 ± 1732	2750 ± 1857	< 0.01	2172 ± 1524	2785 ± 1934	< 0.01
**Mortality**							
In hospital	14.4% (411)	11.7% (273)	26.4% (134)	< 0.01	8.5% (126)	20.6% (280)	< 0.01
28 days (n = 2468)	19.0% (469)	15.2% (308)	36.6% (159)	< 0.01	11.7% (150)	27.1% (316)	< 0.01
Any ICU admission	72.0% (2059)	70.7% (1651)	78.2% (397)	< 0.01	64.7% (956)	80.0% (1090)	< 0.01
Intubated (ED)	8.5% (241)	5.6% (130)	21.0% (106)	< 0.01	3.4% (50)	13.8% (187)	< 0.01
ALI (SF ratio < 452)	58.0% (1659)	53.6% (1252)	77.8% (395)	< 0.01	45.2% (668)	71.5% (974)	< 0.01
Vasopressor(s)	7.2% (205)	5.6% (131)	14.4% (73)	< 0.01	2.5% (37)	12.3% (167)	< 0.01

**Relationship between tqSOFA, qSOFA, lactate and In-Hospital Mortality** tqSOFA was ≥ 2 in 17.9% of patients. Mortality for tqSOFA < 2 was 11.7% and for tqSOFA ≥ 2 mortality was 26.4%. Maximum qSOFA during ED stay was ≥ 2 in 48.0% of the cohort; in-house mortality for patients with maximum qSOFA < 2 was 8.5% and for maximum qSOFA ≥ 2 was 20.6% (Table 1). An initial serum lactate > 3 mmol/L was present in 43.9% of patients presenting after January 1, 2005 (total tested, n = 2413). Mortality was 21.5% in patients with a lactate > 3 mmol/L (Table 2). The sensitivity of tqSOFA for predicting in-hospital mortality was 32.9%. The sensitivity of maximum qSOFA ≥ 2 for predicting in-hospital mortality was 69.0%. The sensitivity of a lactate > 3 mmol/L for predicting in-hospital mortality was 67.8% (Table 3). In this cohort of sepsis patients, lactate and maximum qSOFA performed similarly and each performed better than tqSOFA (AUROC 0.63, 0.62 and 0.59 respectively) (Table 3). Of note, tqSOFA was derived from triage vital signs (taken at ED presentation); the first lactate was drawn at a median of 58 min (IQR 27–123) after presentation; and the maximum qSOFA was derived from the worst vitals during the ED length of stay, with a mean length of stay of 477 (± 248) min.

**Table 2:** Patient Demographics and clinical characteristics stratified by lactate level.

Variables	All patients (n = 2859)	Lactate ≤ 3 (n = 1355)	Lactate > 3 (n = 1058)	*p*-value
Age (yr)	57.3 ± 17.8	56.3 ± 17.7	59.1 ± 17.7	< 0.01
Male	52.9% (1522)	53.7% (727)	54.2% (573)	ns
Time to antibiotic (min)	184 ± 157	189 ± 156	155 ± 138	< 0.01
Total IVF (mL)	2460 ± 1759	2266 ± 1588	3062 ± 1885	< 0.01
**Mortality**				
In hospital	14.4% (411)	8.9% (121)	21.5% (227)	< 0.01
28 days (n = 2468)	19.0% (469)	12.6% (148)	28.3% (261)	< 0.01
Any ICU admission	72.0% (2059)	68.2% (924)	81.1% (858)	< 0.01
Intubated (ED)	8.5% (241)	4.0% (54)	15.3% (161)	< 0.01
ALI (SF ratio < 452)	58.0% (1659)	53.0% (718)	64.7% (684)	< 0.01
Vasopressor(s)	7.2% (205)	4.1% (55)	11.6% (123)	< 0.01

**Table 3:** Accuracy for outcome prediction of in-hospital mortality over different test cut-points.

	Sensitivity (95% CI)	Specificity (95% CI)	NPV (95% CI)	PPV (95% CI)	AUC (95% CI)
tSIRS ≥ 2 (n = 2859)	82.2 (78.2–85.8)	17.7 (16.2–19.3)	85.6 (82.2–88.5)	14.4 (13.0–15.8)	0.50 (0.48–0.52)
maxSIRS ≥ 2 (n = 2859)	98.1 (96.2–99.2)	2.2 (1.8–2.8)	81.4 (66.6–91.6)	14.3 (13.0–15.7)	0.50 (0.49–0.50)
Initial lactate ≥ 3 mmol/L (n = 2306)	67.8 (62.4–72.8)	57.4 (55.2–59.6)	91.5 (89.9–93.0)	20.8 (18.4–23.3)	0.63 (0.60–0.65)
Repeat Lactate ≥ 3 mmol/L (n = 1320)	54.8 (48.4–61.1)	76.1 (73.5–78.6)	87.9 (85.7–90.0)	34.7 (30.0–39.6)	0.65 (0.62–0.69)
Lactate clearance ≥ 10% (n = 1320)	66.1 (59.9–72.0)	17.7 (15.5–20.1)	69.3 (63.5–74.7)	15.7 (13.5–18.0)	0.42 (0.39–0.45)
tqSOFA ≥ 2 (n = 2845)	32.9 (28.4–37.7)	84.7 (83.2–86.1)	88.3 (86.9–89.6)	26.4 (22.6–30.4)	0.59 (0.56–0.61)
qSOFA ≥ 2 (n = 2840)	69.0 (64.2–73.4)	55.5 (53.5–57.5)	91.5 (89.9–92.8)	20.6 (18.4–22.8)	0.62 (0.60–0.65)

Overall mortality rate in severe sepsis dataset: 14.3% (95% CI: 13.0% to 15.6%).

## Discussion

In this analysis of a large single center highly granular database of patients admitted to the hospital with sepsis, triage qSOFA ≥ 2 had low sensitivity for in-hospital mortality and triage qSOFA negative sepsis patients were at significant risk of suffering in-hospital mortality (11.7%). Although the maximum qSOFA performed somewhat better, its utility as a screening tool is limited precisely because it is an overall composite score, resulting from multiple repeat evaluations, and does not serve as an initial predictor, helping to expedite and guide care. Several hours may pass during which a patient evolves from tqSOFA negative to qSOFA positive and during this period care may be delayed.

tqSOFA is calculated at ED triage, the portal of entry for many sepsis patients into the healthcare system, and it performed poorly, identifying a remarkably low percentage (17.9%) of patients who go on to meet criteria for sepsis, require hospital admission, and, more often than not, ICU level care. Further, tqSOFA < 2 mislabeled a significant minority of patients who would ultimately die during their hospitalization (11.7%). In aggregate this suggests that tqSOFA utilized as an early screening tool does not adequately identify the vast majority of at-risk patients and maximum qSOFA, derived from worst values over the course of the ED stay, is neither sufficiently sensitive nor specific. In comparison, a serum lactate value ordered early in the ED course was as predictive of risk for poor outcome as maximum qSOFA but was available on average at 58 min after triage instead of only becoming available over the course of the ED stay. These findings suggest that tqSOFA as an isolated value cannot replace SIRS criteria and that maximum qSOFA calculated from the worse overall variables during ED length of stay does not outperform an early serum lactate.

The SEPSIS-3 task force suggested that one of the main goals of the 2016 sepsis definitions was “to differentiate sepsis from uncomplicated infection^1^.” This implies that patients with sepsis should routinely have a qSOFA score ≥ 2 while those with uncomplicated infection should not. Our data suggest that this is not the case and that a diagnosis of sepsis cannot be achieved simply by applying qSOFA to a population of patients in whom there is a documented or suspected infection and the potential for sepsis. Testing the validity of the qSOFA criteria in a large clinical database, as suggested by the task force, we have demonstrated that qSOFA fails as an early recognition tool: it fails to meet the goal articulated in the SEPSIS-3 definitions that “Patients with suspected infection who are likely to have a prolonged ICU stay or to die in the hospital can be promptly identified at the bedside with qSOFA, i.e., alteration in mental status, systolic blood pressure ≤ 100 mmHg, or respiratory rate ≥ 22/min^1^.”

Since publication of the SEPSIS-3 recommendations, numerous authors have analyzed qSOFA in retrospective and prospective cohorts at different points in the care continuum from pre-hospital to ED to wards to the ICU. For example, in a cohort of 1,045 patients with suspected infection presenting to the ED, Gando and colleagues examined the ability of SIRS vs. qSOFA to predict established infection and demonstrated that SIRS was superior to qSOFA for this distinction (AUROC 0.65 vs. 0.58 respectively)^21^. In contrast, Abdullah et al*.* studied a group of 2,112 infected patients admitted to the ED and obtained data to calculate SIRS and qSOFA scores from the electronic triage data obtained on arrival to the hospital and found both SIRS and qSOFA to have poor sensitivity (52.8% vs. 19.5%, respectively) and divergent specificity (52.5% vs. 92.6%, respectively) for 28-day mortality^24^. Churpek et al*.* compared qSOFA, SIRS criteria, the Modified Early Warning Score, and the National Early Warning Score and found that the early warning scores are more accurate than qSOFA in predicting in-hospital mortality^36^. In addition, the authors found that qSOFA was as predictive as SIRS criteria in identifying patients with a likelihood of adverse outcome. Of note, this study included 30,677 patients with suspicion of infection with 60% identified in the ED setting, while the other 40% were identified as in-patients. Further, Freund et al*.* published results from a multicenter prospective trial exploring the predictive capability of qSOFA to identify ED patients with a high likelihood of in-hospital mortality from sepsis. In this cohort qSOFA performed better than SIRS with AUROCs of 0.80 (95% CI 0.74–0.85) versus 0.65 (95% CI 0.59–0.70), respectively. This study was a prospective analysis of patients who presented to the ED with suspicion of infection. Overall, mortality was low in this cohort (8.4%) and a small sample size was explored (879 patients)^22^. In contrast, our data represents a population of 2,859 sepsis patients with an in-hospital mortality rate over 14%. In a retrospective, three-center study of 928 patients with severe sepsis and septic shock treated in Korean EDs with a 28-day mortality of 24.9%, Kim et al*.* found that 53.1% of the patients and 38.1% of the patients who died had a qSOFA score < 2^18^. They concluded that, in their clinical setting, qSOFA were not sensitive enough to be used as an ED-based screening tool. Finally, in the study most similar to ours, Hwang et al*.* examined the sensitivity of serial qSOFA scores applied at triage, 3, 6, and 24 h after triage to identify patients at risk of death from sepsis. In a retrospective analysis of 1,395 patients diagnosed with severe sepsis or septic shock during their ED stay (sepsis “present on admission”), with an overall 28-day mortality of 15%, tqSOFA had a sensitivity of 39%, a specificity of 77%, and an AUROC of 58%. They observed a similar increasing sensitivity and decreasing specificity as we did as time passed during the ED stay and more patients met qSOFA criteria^20^. Also, like our study demonstrated, patients who met qSOFA criteria at triage were clearly sick, with significantly higher mortality than those who did not meet qSOFA criteria. The problem they found, as did we, was that the tqSOFA negative patients had unacceptably high mortality and other means to identify them are needed.

The SEPSIS-3 authors created a flow diagram (“Operationalization of Clinical Criteria Identifying Patients with Sepsis or Septic Shock”) for the identification of patients with sepsis and recommended the application of qSOFA to help move patients from the suspected infection category to the sepsis category. Viewed schematically, our data strongly suggest that this step should be eliminated because whether qSOFA is <2 or ≥ 2, sepsis must still be suspected and active screening continued instead of shifting into a slower, continuous monitoring mode as suggested by the SEPSIS-3 authors. Thus, the first step in evaluating a “Patient with Suspected Infection” must incorporate other means of deciding whether the patient is at high risk for sepsis. The SEPSIS-3 authors relied on the data from the Kaukonen et al*.* study, which showed that 12.1% of sepsis patients admitted to the ICU didn’t meet SIRS criteria, to eliminate the role of SIRS in sepsis screening. Our data and data from similar studies suggest that this removal was premature. Our data and that from Hwang et al*.* suggest that SIRS is essential to and qSOFA not sufficient for the efficient, timely progression of potential sepsis patients from undifferentiated to partially differentiated to fully differentiated (diagnosed) while treatment is underway. In addition to re-implementing SIRS criteria, these findings emphasize the need for inclusive and continual screening for signs of acute organ dysfunction, even when qSOFA is < 2.

Improvements in the clinical management of sepsis over the past two decades have incorporated early identification, rapid initiation of resuscitation, immediate source control, and early administration of appropriate antibiotics into a multifaceted approach to patients with potentially life-threatening infections. All information that can assist in determining whether a patient has sepsis and is at a high risk for dying in the hospital should be systematically incorporated into early assessment of potential sepsis patients. The goal is to identify potential sepsis patients as soon as possible, begin further assessment and management, further identify those with sepsis as soon as the first acute organ dysfunction attributable to the active infection occurs, and streamline management of their care. *In other words, real-time identification and management of patients with sepsis present on admission to the ED is the ultimate goal.*

**Figure 1:**
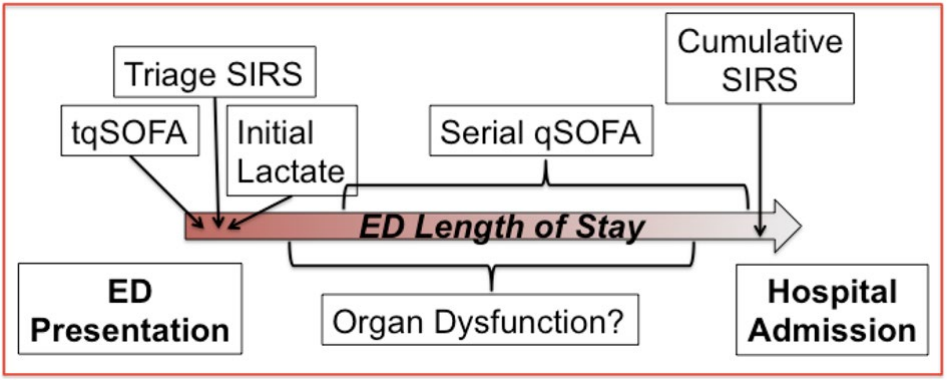
Modified schemata for ED operationalization of clinical and laboratory criteria to detect patients with sepsis.

Additional studies are needed to articulate the optimal screening paradigm across the care continuum. At this point in time, we recommend a stepwise screening approach to patients with potential sepsis, which can begin in the pre-hospital setting if presenting by EMS^16,17,21^ or from a physician’s office or urgent care center, can integrate patient vital signs obtained from home devices, be augmented at triage, and continue to incorporate new data throughout the ED stay. This optimal screening paradigm needs to acknowledge that in initial screening sensitivity is more important than specificity to optimize capture of *potential* patients. Data that should be incorporated should include: triage SIRS, tqSOFA, a serum lactate obtained during or shortly after triage, serial qSOFA over ED stay, and cumulative SIRS criteria over the ED course. It is interesting to note that the SEPSIS-3 authors relied heavily on the results of Kaukonen et al.’s work on SIRS criteria in sepsis patients to argue that SIRS criteria are neither sensitive nor specific enough to be relied upon to capture patients with sepsis since only 87.9% of patients had two or more SIRS criteria on admission to the ICU^14^. In our cohort, 82.2% of patients had two or more SIRS criteria on the vital signs obtained in ED triage and this percentage increased to 98.1% of patients over the course of their ED stay. This suggests that cumulative SIRS criteria will capture essentially all patients with sepsis in the ED and, when augmented by early suspicion of infection, other clinical and laboratory values need to be added to assess in the most efficient and timely manner whether a patient with infection has acute organ dysfunction, risk stratify the patients, and identify those at higher risk for in-hospital mortality (Fig. 1).

This study has several limitations. This is a retrospective analysis of a single center sepsis database and our findings may not be generalizable to other hospitals in different settings. Worst vital sign values incorporated into the qSOFA score were not time-stamped so we were unable to know when in the course of their ED care a patient met qSOFA ≥ 2 or had their maximum qSOFA if these did not occur at triage, which were incorporated into the tqSOFA score. Although the database has triage and worst values for respiratory rate and systolic blood pressure, the only value for mental status in the database was GCS recorded at triage. It is possible that some patients had a decline in mental status during their ED stay and that we are therefore underestimating the percentage of patients with qSOFA ≥ 2 at some point while in the ED. This could underestimate the sensitivity of qSOFA for identifying high-risk patients. Clinical care has changed since the time these data were collected and that may have implications on outcomes and, thus, on sensitivity and specificity of qSOFA values calculated. Use of lactate as a screening tool has increased and this increase may change its sensitivity and specificity. Finally, some patients with qSOFA < 2 were transferred rapidly from the ED to medical wards and the ICU and it is possible that they met qSOFA ≥ 2 criteria shortly after leaving the ED. Again, this might underestimate the sensitivity of the qSOFA score.

In conclusion, in a large ED sepsis database, tqSOFA performed poorly as a means to identify patients at increased risk of mortality on initial presentation. In addition, maximum qSOFA during the ED stay did not outperform initial serum lactate levels. These data suggest that qSOFA cannot be relied upon to identify sepsis patients at high risk of death and that other means of rapidly identifying high-risk patients are needed in settings where undifferentiated patients present for emergent care.

## Data Availability

The database is available with reasonable notification to other researchers by contacting the corresponding author.
